# Melatonin Enhances the Photosynthesis and Antioxidant Enzyme Activities of Mung Bean under Drought and High-Temperature Stress Conditions

**DOI:** 10.3390/plants12132535

**Published:** 2023-07-03

**Authors:** Anitha Kuppusamy, Senthil Alagarswamy, Kalarani M. Karuppusami, Djanaguiraman Maduraimuthu, Senthil Natesan, Kuttimani Ramalingam, Umapathi Muniyappan, Marimuthu Subramanian, Selvaraju Kanagarajan

**Affiliations:** 1 Department of Crop Physiology, Tamil Nadu Agricultural University, Coimbatore 641003, India; niviani95@gmail.com (A.K.); kalarani.mk@tnau.ac.in (K.M.K.); jani@tnau.ac.in (D.M.); kuttimanir@gmail.com (K.R.); umapathi182@gmail.com (U.M.); 2 Centre for Plant Molecular Biology, Tamil Nadu Agricultural University, Coimbatore 641003, India; senthil_natesan@tnau.ac.in; 3 Department of Agronomy, Agricultural College & Research Institute, Eachangkottai, Thanjavur 614904, India; sm20@tnau.ac.in; 4 Department of Plant Breeding, Swedish University of Agricultural Sciences, P.O. Box 190, 23422 Lomma, Sweden

**Keywords:** antioxidant enzyme, drought and high-temperature stress, gas exchange parameters, melatonin, yield

## Abstract

Mung bean, a legume, is sensitive to abiotic stresses at different growth stages, and its yield potential is affected by drought and high-temperature stress at the sensitive stage. Melatonin is a multifunctional hormone that plays a vital role in plant stress defense mechanisms. This study aimed to evaluate the efficiency of melatonin under individual and combined drought and high-temperature stress in mung bean. An experiment was laid out with five treatments, including an exogenous application of 100 µM melatonin as a seed treatment, foliar spray, and a combination of both seed treatment and foliar spray, as well as absolute control (ambient condition) and control (stress without melatonin treatment). Stresses were imposed during the mung bean’s reproductive stage (31–40 DAS) for ten days. Results revealed that drought and high-temperature stress significantly decreased chlorophyll index, Fv/Fm ratio, photosynthetic rate, stomatal conductance, and transpiration rate through increased reactive oxygen species (ROS) production. Foliar application of melatonin at 100 µM concentration enhanced the activity of antioxidant enzymes such as superoxide dismutase, catalase, and ascorbate peroxidase and the concentration of metabolites involved in osmoregulation and ion homeostasis; thereby, it improves physiological and yield-related traits in mung bean under individual and combined stress at the reproductive stage.

## 1. Introduction

Pulses provide a vital source of proteins, minerals, and nutrients for millions living below the poverty line in developing countries. The Indian Council of Medical Research (ICMR) has suggested a minimum consumption of 40 g of pulses per day, while the World Health Organization (WHO) advises 100 g [1]. The production of pulses has to meet the recommendation of ICMR; still, the production needs to be increased to achieve the target of the WHO. The current level of pulse production needs to be doubled by minimizing the gap between supply and demand by growing pulses in nutrient-rich, fertile soils.

Among pulses, mung bean (*Vigna radiata* L.) is the third most important leguminous crop next to chickpea and pigeon pea. Mung bean is popularly known as green gram and is cultivated in India and other Asian countries. It is a short-duration annual crop. It improves soil nitrogen levels by fixing atmospheric nitrogen by creating a symbiotic relationship with microorganisms like Rhizobium and plays a major role in sustainable agriculture. The low productivity of mung bean may be due to the cultivation of crops in marginal and sub-marginal soil, poor management practices, inadequate rainfall, insufficient use of fertilizers, and pest and disease attack [2].

Mung bean requires an appropriate quantity of water and optimum temperature for higher yields. The optimum temperature for mung bean cultivation ranges from 25 to 30 °C [3]. The maximum temperature during the summer usually exceeds 40 °C, which can severely affect the mung bean growth and yield [4]. Drought stress has been reported to affect around 68 percent of India’s net cultivated land (140 million hectares), and a significant reduction in soil moisture level reduces crop yield [5]. According to the IPCC [6] assessment, an increase in temperature and water scarcity causes a significant impact on agricultural productivity that affects the quality and yield of the crop [7].

Water stress alters plant water status and affects numerous physiological processes like photosynthesis, translocation of photosynthates, nutrient absorption, ion uptake, and the activity of several enzymes also found to decline in response to cell desiccation [8]. High temperature disrupts synthesis, appropriate folding, and stability of proteins and causes enzyme inactivation that increases cellular fluidity due to the production of reactive oxygen species (ROS) and reactive nitrogen species (RNS) [9], which all lead to a negative impact on plant growth. Drought and high-temperature stress during the crop’s reproduction stage affects pollen and stigma viability, pollen tube growth, fertilization, and embryogenesis [10].

Several management options have been tried with nutrients, plant growth regulators (PGRs), and chemicals to alleviate the adverse effects of drought and high temperature on yield. Plant growth regulators are multifunctional compounds with well-established physiological roles in plants. One such growth-regulating natural compound is “melatonin”, which can alleviate the adverse effects of abiotic stresses. As melatonin is an anti-stressor compound, it protects the plant from the harmful effects of oxidative stress induced by drought and high-temperature stress [11].

Melatonin is structurally related to tryptophan, and its biosynthesis is analogous to that of auxin, serotonin, and its isoforms [12]. It acts as an antioxidant and detoxifies ROS and RNS by enhancing the antioxidant enzyme activity under stress conditions [13]. Melatonin act as a promoter of seed germination [14] and lateral root formation [15], regulates flowering [16], and delays leaf senescence [17] to manage and control plant growth and development under challenging environments.

Melatonin is an endogenous substance associated with seed germination, root growth, pigment content, photorespiration, stomatal conductance, water use efficiency, and seed yield [18]. Melatonin also protects the ultrastructure of chloroplasts from damage. It promotes the efficiency of PSII, carbon fixation, and the electron transport system, which results in enhanced photosynthetic processes under different abiotic stresses [19,20,21]. Exogenous melatonin can stimulate the biosynthesis of endogenous melatonin, reduce H_2_O_2_, O_2_^−^, and malondialdehyde generation under various abiotic stresses, and improve the photosynthetic process [22].

Exogenous melatonin improves the soluble protein, proline, and relative water content of the plant under drought stress, which may aid in maintaining the turgor pressure and integrity of the cell [23]. The activities of enzymatic antioxidants such as superoxide dismutase (SOD), catalase (CAT), ascorbate peroxidase (APX), and glutathione reductase (GR) and non-enzymatic antioxidants like ascorbate, glutathione, and carotenoid were increased by melatonin in response to various environmental stresses [24] that minimized the production of ROS and RNS and protected the plant from cell membrane damage through reduced lipid peroxidation [25]. The synthesis and accumulation of primary and secondary metabolites like carbohydrates, proteins, lipids, and phenols were increased by melatonin application, which improves the plant’s osmotic adjustment and water status under abiotic stresses [26]. Melatonin increases the growth-related attributes by stimulating the seed germination process, maintaining a robust root system, and improving photosynthetic capacity by delaying leaf senescence, contributing to enhanced yield and yield characteristics under stress conditions [27]. However, little information and research were available on melatonin-mediated regulation of abiotic stress tolerance in pulses, especially mung bean, under combined stress like drought and high temperature. Considering these aspects, the current study has been conducted to increase mung bean production under drought and high-temperature stress.

## 2. Results

### 2.1. Effect of Melatonin on Physiological, Biochemical, and Yield Characteristics of Mung Bean under Drought Stress

**Figure 1:**
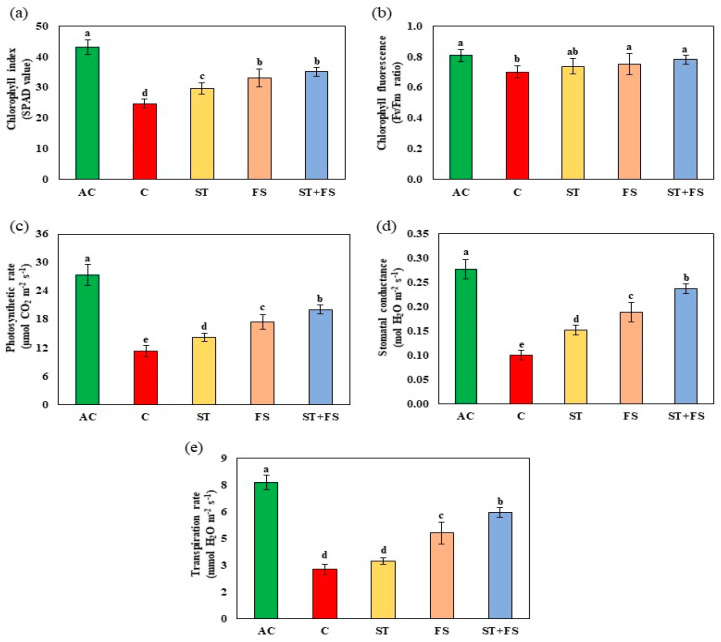
Impact of melatonin on (**a**) chlorophyll index, (**b**) Fv/Fm ratio, (**c**) photosynthetic rate, (**d**) stomatal conductance, and (**e**) transpiration rate of mung bean under drought stress conditions. AC—absolute control (green); C—control (red); ST—seed treatment of 100 µM melatonin (yellow); FS—foliar spray of 100 µM melatonin (brown); ST + FS—seed treatment plus a foliar spray of 100 µM melatonin (blue). Least significant difference test was used to compare the differences among group means, and the critical difference was computed at *p* ≤ 0.05. Values with different letters are significantly different (*n* = 4).

The physiological parameters such as chlorophyll index, Fv/Fm ratio, photosynthetic rate, stomatal conductance, and transpiration rate were significantly (*p* < 0.05) reduced in drought-stressed control plants. Applying 100 µM of melatonin enhanced the physiological activity of mung bean under drought stress (Figure 1a–e). Combined seed treatment and foliar spray of 100 µM melatonin increased the chlorophyll index, Fv/Fm ratio, photosynthetic rate, stomatal conductance, and transpiration rate by 42%, 12%, 43%, 52%, and 53%, respectively, compared to control plants.

**Figure 2:**
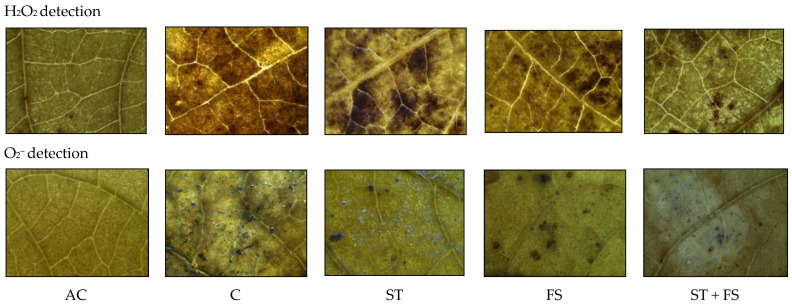
In situ detection of H_2_O_2_ and O_2_^−^ production by histochemical analysis in mung bean leaves exposed to drought stress. AC—absolute control; C—control; ST—seed treatment of 100 µM melatonin; FS—foliar spray of 100 µM melatonin; ST + FS—seed treatment plus a foliar spray of 100 µM melatonin. Representative images in each treatment are presented.

Histochemical analysis of the production of ROS like hydrogen peroxide (H_2_O_2_) and superoxide (O_2_^−^) in mung bean leaves exposed to drought stress is presented in Figure 2. Under irrigated conditions, H_2_O_2_ and O_2_^−^ radical generation and accumulation were negligible in mung bean leaf tissues. In contrast, the plants subjected to drought stress showed a significant increase in H_2_O_2_ and O_2_^−^ production in leaf tissues of mung bean, which is indicated by brown and blue staining. The application of melatonin significantly reduced the concentration of H_2_O_2_ and O_2_^−^ compared to drought-stressed control plants. Among the melatonin treatments, seed treatment plus the foliar application of 100 µM melatonin showed minimum H_2_O_2_ and O_2_^−^ accumulation in leaf tissues and exhibited less brown and blue staining in mung bean leaves than other leaves melatonin treatments at the reproductive stage under drought stress.

**Figure 3:**
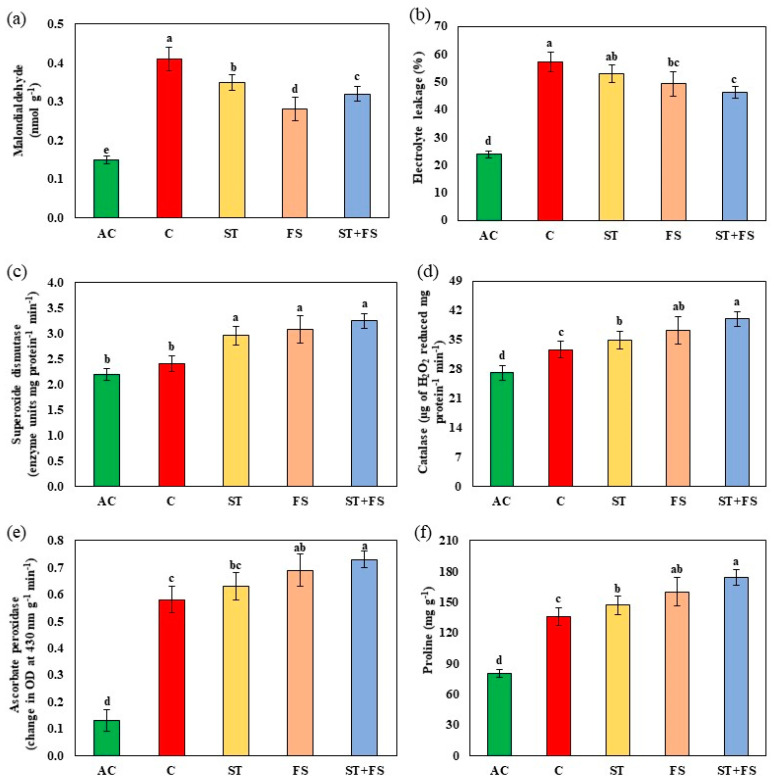
Impact of melatonin on (**a**) malondialdehyde content, (**b**) electrolyte leakage, (**c**) superoxide dismutase, (**d**) catalase, (**e**) ascorbate peroxidase, and (**f**) proline of mung bean under drought stress conditions. AC—absolute control (green); C—control (red); ST—seed treatment of 100 µM melatonin (yellow); FS—foliar spray of 100 µM melatonin (brown); ST + FS—seed treatment plus a foliar spray of 100 µM melatonin (blue). Least significant difference test was used to compare the differences among group means, and the critical difference was computed at *p* ≤ 0.05. Values with different letters are significantly different (*n* = 4).

The results of the malondialdehyde and electrolyte leakage assay, which indicates membrane damage, was high in control plants subjected to drought stress, as shown in Figure 3a,b. In contrast, the melatonin-treated plants exhibited significantly (*p* < 0.05) reduced membrane damage under drought stress during the reproductive stage of mung bean. Combined application of 100 µM melatonin as seed treatment and foliar spray reduced malondialdehyde and electrolyte leakage by about 22% and 20%, respectively, compared to control plants.

The activity of antioxidant enzymes such as superoxide dismutase, catalase, and ascorbate peroxidase were enhanced in melatonin-treated plants compared to control plants under drought stress (Figure 3c–e). Increases of about 35%, 23%, and 26% in the activity of superoxide dismutase, catalase, and ascorbate peroxidase were observed in the foliar spray of 100 µM melatonin as a combination of both seed treatment and foliar spray under drought stress in mung bean during the reproductive stage. Proline was recorded nearly 28% more during drought stress after the exogenous application of melatonin at 100 µM as combined seed treatment and foliar spray compared to control plants (Figure 3f).

**Figure 4:**
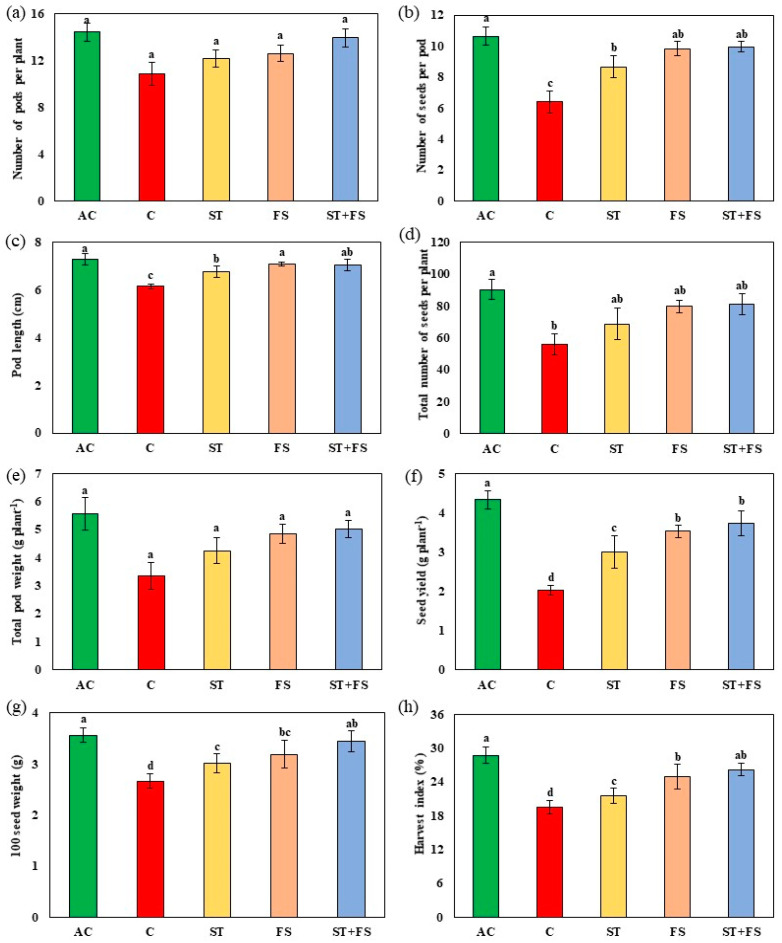
Impact of melatonin on (**a**) number of pods per plant, (**b**) number of seeds per pod, (**c**) pod length, (**d**) total number of seeds per plant, (**e**) total pod weight, (**f**) seed yield per plant, (**g**) 100 seed weight, and (**h**) harvest index of mung bean under drought stress conditions. AC—absolute control (green); C—control (red); ST—seed treatment of 100 µM melatonin (yellow); FS—foliar spray of 100 µM melatonin (brown); ST + FS—seed treatment plus a foliar spray of 100 µM melatonin (blue). Least significant difference test was used to compare the differences among group means, and the critical difference was computed at *p* ≤ 0.05. Values with different letters are significantly different (*n* = 4).

Drought stress reduced the yield to about 53% in control plants compared to the irrigated plants (Figure 4). The combined effect of seed treatment and foliar application of 100 µM melatonin increased the number of pods per plant by 28%, the number of seeds per pod by 55%, pod length by 14%, the total number of seeds per plant by 45%, total pod weight by 50%, seed yield per plant by 74%, 100 seed weight by 30%, and harvest index by 34% under drought stress when compared to control plants.

### 2.2. Effect of Melatonin on Physiological, Biochemical, and Yield Characteristics of Mung Bean under High-Temperature Stress

**Figure 5:**
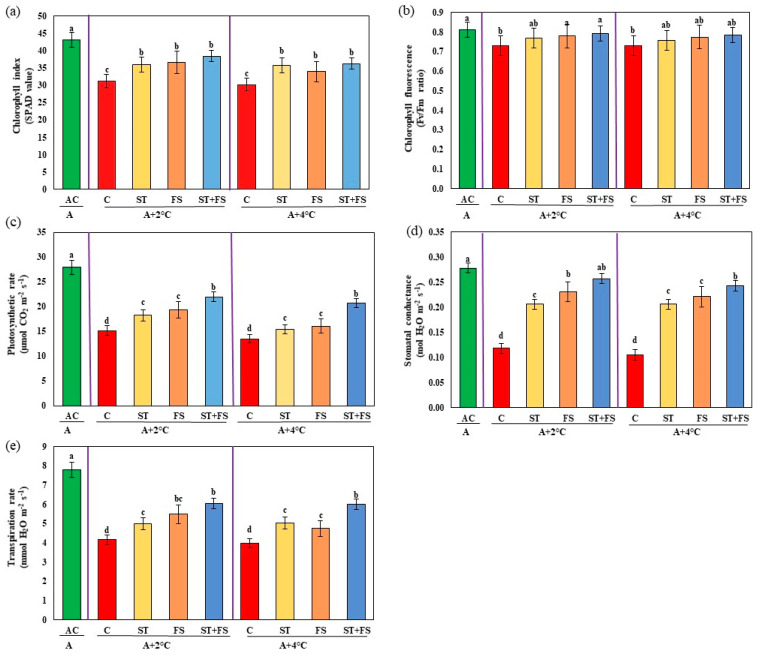
Impact of melatonin on (**a**) chlorophyll index, (**b**) Fv/Fm ratio, (**c**) photosynthetic rate, (**d**) stomatal conductance, and (**e**) transpiration rate of mung bean under high-temperature stress conditions. AC—absolute control (green); C—control (red); ST—seed treatment of 100 µM melatonin (yellow); FS—foliar spray of 100 µM melatonin (brown); ST + FS—seed treatment plus a foliar spray of 100 µM melatonin (blue). Least significant difference test was used to compare the differences among group means, and the critical difference was computed at *p* ≤ 0.05. Values with different letters are significantly different (*n* = 4).

Foliar spray of 100 µM melatonin at 34 °C and 36 °C increased the chlorophyll index by 23% and 20% and Fv/Fm ratio by 7% and 8% over unsprayed control (Figure 5). Similarly, photosynthetic rate, stomatal conductance, and transpiration rate were increased by foliar spray of melatonin at 100 µM as combined seed treatment and foliar spray during reproductive stage stress. About 45% and 54% increases in photosynthetic rate, 54% and 56% increases in stomatal conductance, and 45% and 51% increases in transpiration rate were observed at 34 °C and 36 °C of high-temperature stress, respectively, compared to the control.

**Figure 6:**
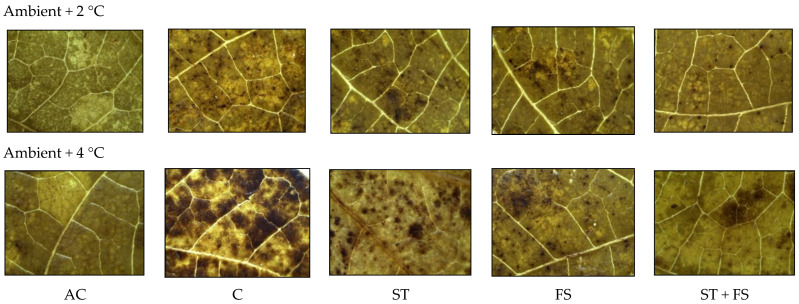
In situ detection of H_2_O_2_ production by histochemical analysis in mung bean leaves exposed to high-temperature stress. AC—absolute control; C—control; ST—seed treatment of 100 µM melatonin; FS—foliar spray of 100 µM melatonin; ST + FS—seed treatment plus a foliar spray of 100 µM melatonin.

**Figure 7:**
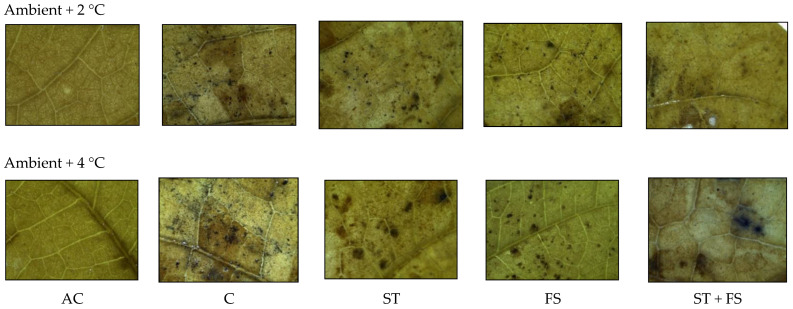
In situ detection of O_2_^−^ production by histochemical analysis in mung bean leaves exposed to high-temperature stress. AC—absolute control; C—control; ST—seed treatment of 100 µM melatonin; FS—foliar spray of 100 µM melatonin; ST + FS—seed treatment plus a foliar spray of 100 µM melatonin.

The generation of H_2_O_2_ and O_2_^−^ in mung bean leaves under high-temperature stress was examined histochemically (Figure 6 and Figure 7). Compared to control plants, plants that were treated with 100 µM of melatonin showed reduced accumulation of H_2_O_2_ and O_2_^−^ in leaf tissues exposed to 34 °C and 36 °C of high-temperature stress, as shown by the reduced intensity of brown and blue colors.

**Figure 8:**
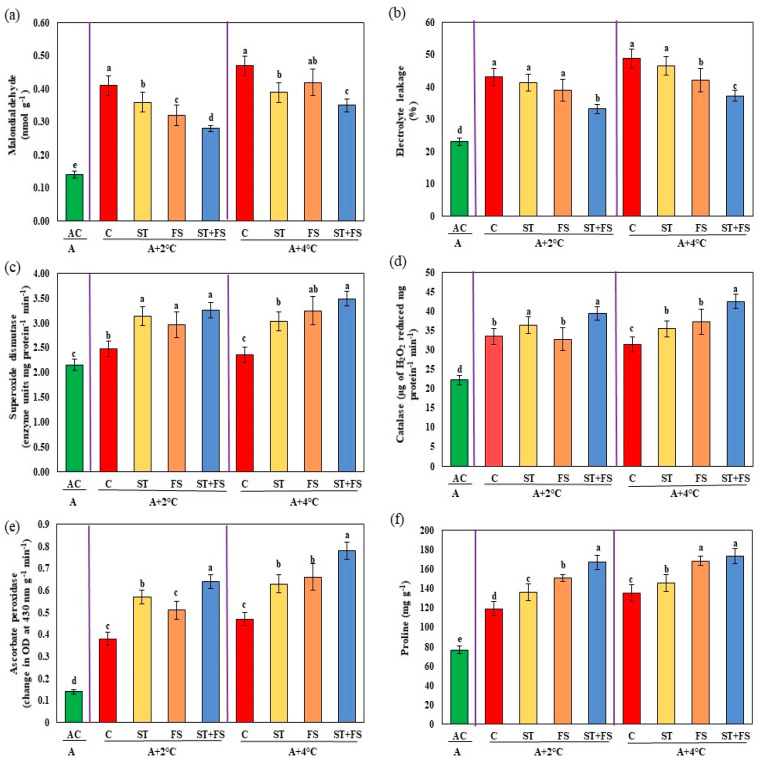
Impact of melatonin on (**a**) malondialdehyde content, (**b**) electrolyte leakage, (**c**) superoxide dismutase, (**d**) catalase, (**e**) ascorbate peroxidase, and (**f**) proline of mung bean under high-temperature stress. AC—absolute control (green); C—control (red); ST—seed treatment of 100 µM melatonin (yellow); FS—foliar spray of 100 µM melatonin (brown); ST + FS—seed treatment plus a foliar spray of 100 µM melatonin (blue). Least significant difference test was used to compare the differences among group means, and the critical difference was computed at *p* ≤ 0.05. Values with different letters are significantly different (*n* = 4).

Exposure to high-temperature stress significantly (*p* < 0.05) increased the malondialdehyde and electrolyte leakage of mung bean exposed to 34 °C and 36 °C (Figure 8a,b). Foliar spray of melatonin at 100 µM concentration as a seed treatment and foliar spray decreased the malondialdehyde by 32% and 26% and electrolyte leakage by 23% and 24% at 34 °C and 36 °C, respectively, compared to the untreated plants.

Application of melatonin at 100 µM as combined seed treatment and foliar spray to mung bean plants significantly (*p* < 0.05) increased the activity of antioxidant enzymes such as superoxide dismutase by 31% and 48%, catalase by 17% and 35%, ascorbate peroxidase by 68% and 66%, and proline by 40% and 28% for 34 °C and 36 °C, respectively, compared to the control plants (Figure 8c–f).

**Figure 9:**
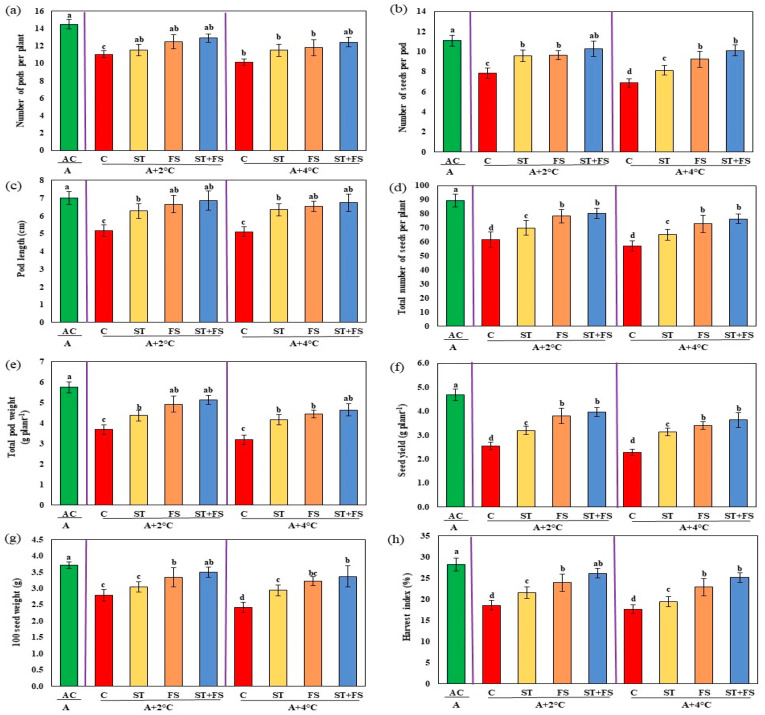
Impact of melatonin on (**a**) number of pods per plant, (**b**) number of seeds per pod, (**c**) pod length, (**d**) total number of seeds per plant, (**e**) total pod weight, (**f**) seed yield per plant, (**g**) 100 seed weight, and (**h**) harvest index of mung bean under high-temperature stress. AC—absolute control (green); C—control (red); ST—seed treatment of 100 µM melatonin (yellow); FS—foliar spray of 100 µM melatonin (brown); ST + FS—seed treatment plus a foliar spray of 100 µM melatonin (blue). Least significant difference test was used to compare the differences among group means, and the critical difference was computed at *p* ≤ 0.05. Values with different letters are significantly different (*n* = 4).

Compared to the plants grown under ambient conditions, the yield of unsprayed plants exposed to high-temperature stress was significantly reduced by about 45% at 34 °C and 51% at 36 °C (Figure 9). Application of 100 µM melatonin as combined seed treatment and foliar spray under 34 °C and 36 °C increased the number of pods per plant (17% and 22%), number of seeds per pod (31% and 47%), pod length (33% and 32%), and total number of seeds per plant (30% and 34%) compared to the unsprayed control plants. The same trend was noticed for pod weight, seed yield per plant, 100 seed weight, and harvest index. Melatonin-treated plants exposed to 34 °C and 36 °C exhibited increases in pod weight by 39% and 46%, 100 seed weight by 25% and 39%, and harvest index by 41% and 43%, respectively, compared to control plants. Compared to control plants, melatonin-treated plants exhibited significantly increased seed yield per plant by nearly 55% and 59%, respectively, at 34 °C and 36 °C.

### 2.3. Effect of Melatonin on Physiological, Biochemical, and Yield Characteristics of Mung Bean under Combined Drought and High-Temperature Stress

**Figure 10:**
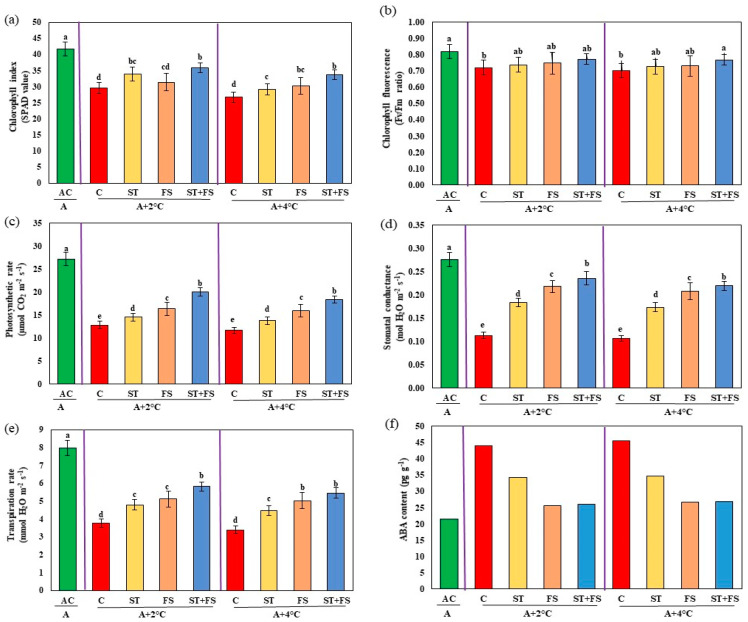
Impact of melatonin on (**a**) chlorophyll index, (**b**) Fv/Fm ratio, (**c**) photosynthetic rate, (**d**) stomatal conductance, (**e**) transpiration rate, and (**f**) ABA content of mung bean under combined drought and high-temperature stress. AC—absolute control (green); C—control (red); ST—seed treatment of 100 µM melatonin (yellow); FS—foliar spray of 100 µM melatonin (brown); ST + FS—seed treatment plus a foliar spray of 100 µM melatonin (blue). Least significant difference test was used to compare the differences among group means, and the critical difference was computed at *p* ≤ 0.05. Values with different letters are significantly different (*n* = 4).

Application of melatonin at 100 µM as combined seed treatment and foliar spray enhanced chlorophyll index by 21% and 26% and Fv/Fm ratio by 7% and 9% under 36 °C and 38 °C, respectively (Figure 10). Application of melatonin at 100 µM as combined seed treatment and foliar spray significantly (*p* < 0.05) increased photosynthetic rate by 56% and 57%, stomatal conductance by 51% and 52%, and transpiration rate by 55% and 61%, respectively, at 36 °C and 38 °C compared to control plants. Plants treated with melatonin exhibited reduced abscisic acid (ABA) levels under drought and high-temperature stress compared to control plants.

**Figure 11:**
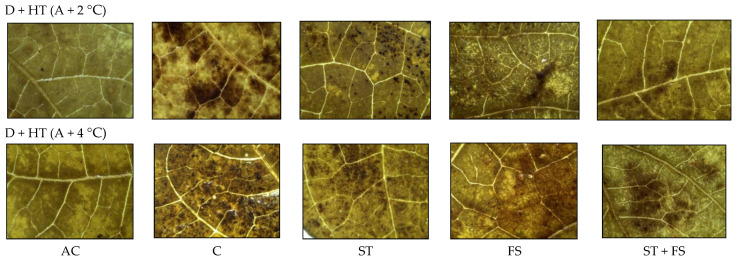
In situ detection of H_2_O_2_ production by histochemical analysis in mung bean leaves exposed to combined drought and high-temperature stress. AC—absolute control; C—control; ST—seed treatment of 100 µM melatonin; FS—foliar spray of 100 µM melatonin; ST + FS—seed treatment plus a foliar spray of 100 µM melatonin.

**Figure 12:**
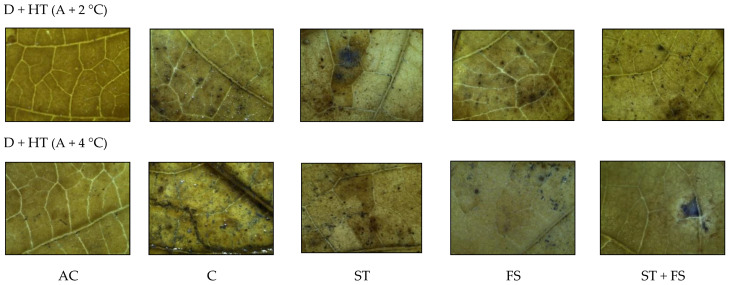
In situ detection of O_2_^−^ production by histochemical analysis in mung bean leaves exposed to combined drought and high-temperature stress. AC—absolute control; C—control; ST—seed treatment of 100 µM melatonin; FS—foliar spray of 100 µM melatonin; ST + FS—seed treatment plus a foliar spray of 100 µM melatonin.

From the histochemical analysis, it was observed that a significant variation in the production of ROS, such as H_2_O_2_ and O_2_^−^, was recorded under combined drought and high-temperature stress (Figure 11 and Figure 12). Melatonin-untreated plants exposed to combined drought and high-temperature stress at 36 °C and 38 °C showed a significant increase in H_2_O_2_ and O_2_^−^ accumulation which was evident from the brown and blue staining intensity in the stressed leaves of mung bean. 

**Figure 13:**
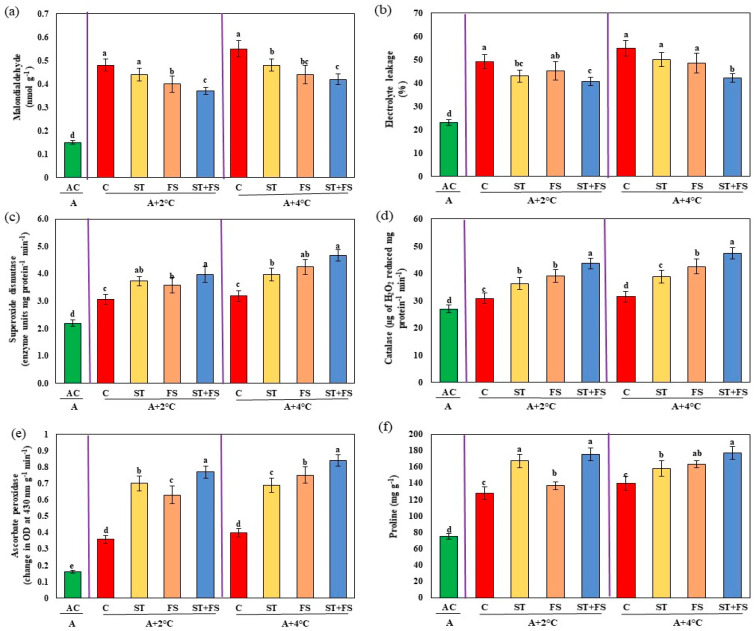
Impact of melatonin on (**a**) malondialdehyde content, (**b**) electrolyte leakage, (**c**) superoxide dismutase, (**d**) catalase, (**e**) ascorbate peroxidase, and (**f**) proline of mung bean under combined drought and high-temperature stress. AC—absolute control (green); C—control (red); ST—seed treatment of 100 µM melatonin (yellow); FS—foliar spray of 100 µM melatonin (brown); ST + FS—seed treatment plus a foliar spray of 100 µM melatonin (blue). Least significant difference test was used to compare the differences among group means, and the critical difference was computed at *p* ≤ 0.05. Values with different letters are significantly different (*n* = 4).

Malondialdehyde and electrolyte leakage were higher in combined drought and high-temperature stress at 36 °C and 38 °C, as shown in Figure 13a,b. Malondialdehyde levels were reduced by 23% and 24% and electrolyte leakage was reduced by 17% and 23% under combined drought and high-temperature stress of 36 °C and 38 °C, respectively, upon application of 100 µM melatonin as seed treatment and foliar spray when compared to melatonin-untreated plants.

Under combined drought and high-temperature stress, melatonin-treated plants had higher levels of activity of antioxidant enzymes such as superoxide dismutase, catalase, and ascorbate peroxidase than melatonin-untreated plants (Figure 13c–e). Combined application of 100 µM melatonin as seed treatment and foliar spray significantly (*p* < 0.05) enhanced superoxide dismutase by 30% and 47%, catalase by 41% and 50%, and ascorbate peroxidase by 52% and 53% under drought and high-temperature stress at 36 °C and 38 °C, respectively, over control plants. Similarly, increased proline content of about 38% and 27% was observed in combined seed treatment and foliar application of 100 µM melatonin under combined drought and high temperatures at 36 °C and 38 °C (Figure 13f).

**Figure 14:**
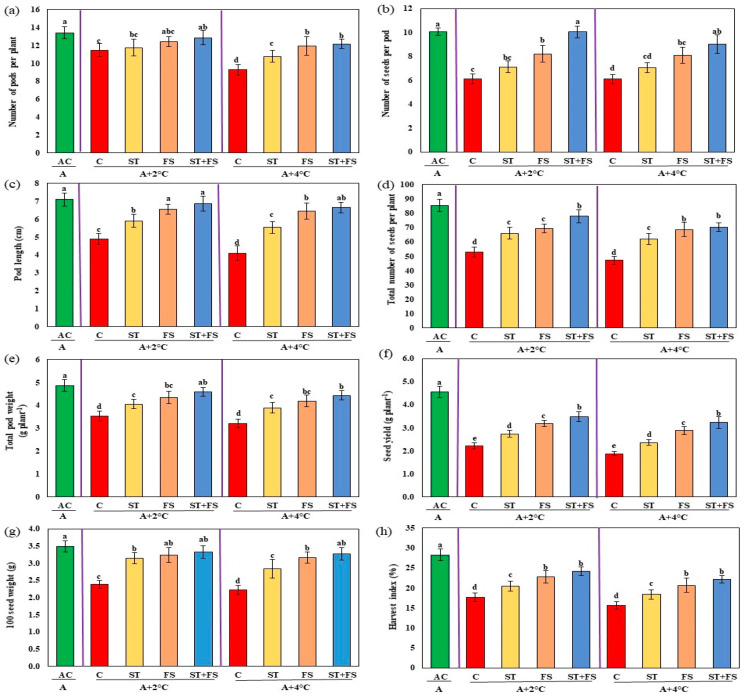
Impact of melatonin on (**a**) number of pods per plant, (**b**) number of seeds per pod, (**c**) pod length, (**d**) total number of seeds per plant, (**e**) total pod weight, (**f**) seed yield per plant, (**g**) 100 seed weight, and (**h**) harvest index of mung bean under combined drought and high-temperature stress. AC—absolute control (green); C—control (red); ST—seed treatment of 100 µM melatonin (yellow); FS—foliar spray of 100 µM melatonin (brown); ST + FS—seed treatment plus a foliar spray of 100 µM melatonin (blue). Least significant difference test was used to compare the differences among group means, and the critical difference was computed at *p* ≤ 0.05. Values with different letters are significantly different (*n* = 4).

**Figure 15:**
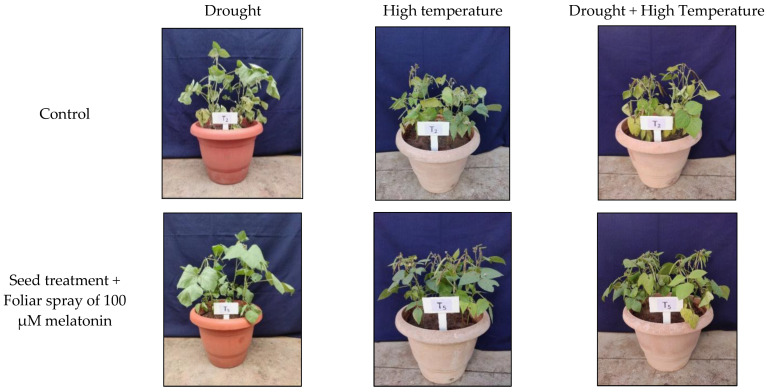
Effect of melatonin on mung bean exposed to individual and combined stresses of drought and high temperature.

The seed yield of control plants (combined drought and high-temperature stress) decreased by around 51% at 36 °C and 58% at 38 °C compared to plants under ambient conditions (Figure 14). A combination of seed treatment and foliar application of 100 µM melatonin increased the number of pods per plant by 12% and 31%, the number of seeds per pod by 63% and 48%, pod length by 40% and 63%, the total number of seeds per plant by 47% and 49%, total pod weight by 30% and 38%, seed yield per plant by 57% and 72%, 100 seed weight by 40% and 47%, and harvest index by 37% and 41% in plants exposed to 36 °C and 38 °C, respectively, under combined drought and high-temperature stress in mung bean (Figure 15).

### 2.4. Effect of Melatonin Treatment on Metabolomics Profiling of Mung Bean under Drought and High-Temperature Stress

**Figure 16:**
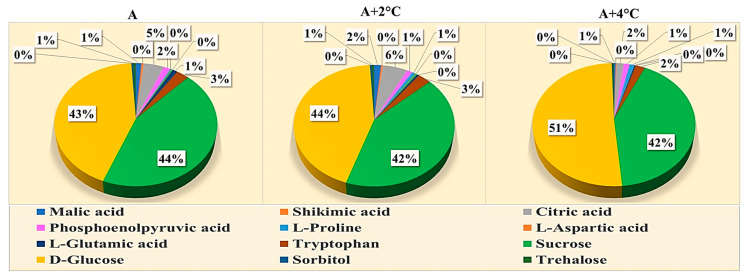
Profiling of metabolites present in mung bean leaves exposed to combined drought and high-temperature stress by GC-MS analysis.

The result of metabolite profiling of leaves exposed to combined drought and high-temperature stress is presented in Figure 16. A total of 13 metabolites were identified with their recognized identity in melatonin untreated and treated mung bean leaves under drought and high-temperature stress. The metabolites comprise five organic acids, four amino acids, two sugars, and two sugar alcohols.

Several metabolites synthesized in melatonin-treated plants were also observed in control plants. However, their peak and area of expression were significantly reduced in control plants compared to melatonin-treated plants under combined drought stress and high temperature at 36 °C and 38 °C. Combined seed treatment plus a foliar application increased the accumulation of amino acids (proline, aspartic acid, glutamic acid, and tryptophan), sugars (sucrose and glucose), organic acids (succinic acid, malic acid, shikimic acid, citric acid, and phosphoenol pyruvic acid), and sugar alcohols (sorbitol and trehalose) over control plants under drought and high-temperature stress of 36 °C and 38 °C.

### 2.5. Effect of Melatonin Treatment on Differentially Expressed Genes of Mung Bean Leaves Exposed to Combined Drought and High-Temperature Stress

**Figure 17:**
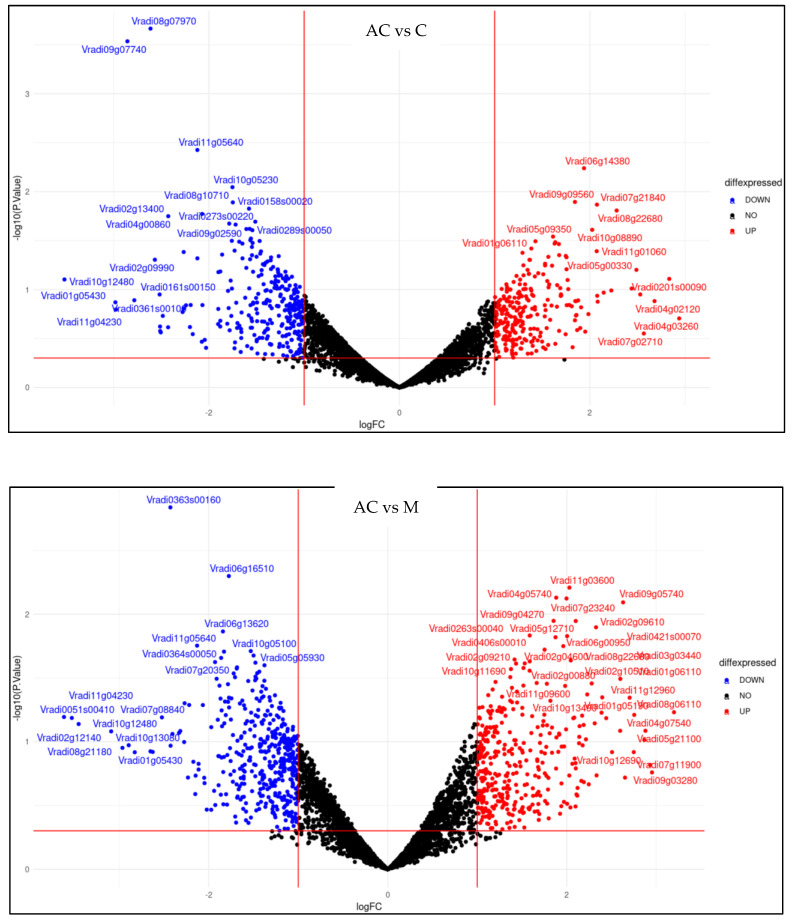

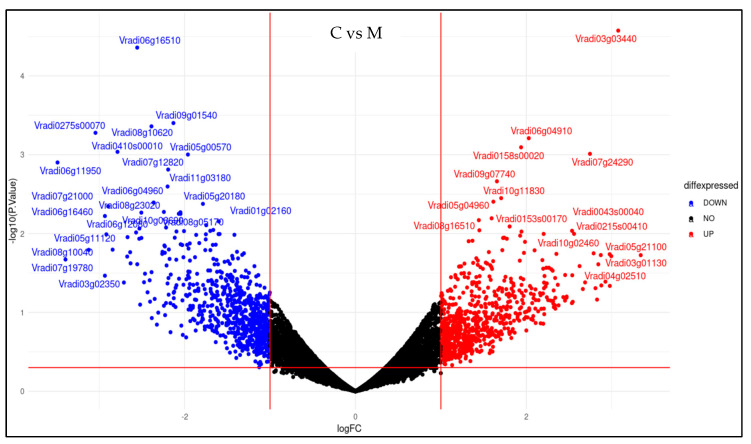
Volcano plot of DEGs between absolute control (AC), control (C), and seed treatment plus foliar spray of 100 µM of melatonin (M) treatments exposed to combined drought and high−temperature stress.

The distribution of differentially expressed genes (DEGs) among the various comparison groups like AC vs. C, AC vs. M, and C vs. M was evaluated. Based on the log fold change (|log_2 FC| ≥ 2), the top five expressed genes and their functions are listed in Figure 17.

## 3. Discussion

Results indicated that abiotic stresses like drought, high temperature, and combined drought and high temperature decreased the traits associated with photosynthetic rate. The abiotic stresses reduced the chlorophyll molecule content, which could indicate chloroplast ultrastructure degradation [28]. In contrast, seed treatment and foliar application of melatonin increased the chlorophyll content compared to unsprayed control, indicating that melatonin could maintain the ultrastructure of chloroplasts under abiotic stresses [29,30,31,32].

The integrity and function of chloroplasts were assessed using chlorophyll fluorescence. Drought, high temperature, or combined drought and high-temperature stress decreased the quantum yield of PSII (Fv/Fm ratio) compared to the absolute control. However, with foliar application of melatonin under drought, high temperature, or combined stresses, the Fv/Fm ratio was higher than that of the unsprayed control, and this could be associated with decreased or repaired photooxidative damage and improved electron transport rate [33,34,35,36].

Photosynthesis is one of the most important physiological processes, and it is severely affected by individual or combined drought and high-temperature stress [37]. The decrease is associated with decreased stomatal conductance and increased abscisic acid content [38]. Foliar application of melatonin under drought, high temperature, or combined drought and high-temperature stress increased the stomatal conductance and decreased the ABA content; thereby, the photosynthetic rate was sustained under stresses [38,39,40]. In melatonin-sprayed plants, the decreased ABA level might have reduced the production of H_2_O_2_ in guard cells, which may keep the stomata open and maintain the plant’s photosynthetic rate under abiotic stresses [41,42,43,44,45]. This study also proved that the application of melatonin increased the expression of photosynthesis-associated proteins like *LHCa*, *PsbA*, *PsbB*, *PsbD*, and *PetE* under stresses, which might have been involved in enhanced photosynthetic rate [42].

Rapid ROS accumulation under drought and high-temperature stress might have decreased the membrane fluidity and altered ion homeostasis [46,47]. Under drought, high temperature, or combined drought and high-temperature stress conditions, the plants had an imbalance between ROS scavenging and antioxidant defense systems [48,49]. In the present study, the unsprayed plants had more membrane damage than the melatonin-sprayed plants, indicating that melatonin could have scavenged the ROS, maintained the membrane integrity, and increased the antioxidant enzyme activity [50,51,52]. In the present study, enhanced antioxidant enzyme activity reduced ROS production and accumulation, electrolyte leakage, and malondialdehyde content in plants under drought and high-temperature stress [53]. 

Proline is one of the compatible solutes that accumulate in plant cells in response to drought stress and increases the osmotic adjustment potential [54]. The enhanced proline content in melatonin-sprayed plants could be due to enhanced biosynthesis [55]. Proline can also act as an antioxidant that protects the plant cell membrane from ROS-induced damage [56]. Sheikhalipour et al. [57] showed that an increase in proline concentration due to melatonin treatment also increases the protection of protein structures from denaturation under drought stress. The results of the current investigation also showed similar findings that enhanced proline content improves plant water status through increased transpiration rate and stomatal conductance in melatonin-treated mung bean plants subjected to drought and high-temperature stress.

Metabolomics profiling of mung bean leaves subjected to combined drought and high-temperature stress revealed that metabolites involved in osmotic adjustment, ion homeostasis, and carbon and amino acid metabolism were upregulated by the exogenous application of melatonin. Saddhe et al. [58] described that metabolites like proline and some sugars such as glucose, fructose, sucrose, and trehalose regulated osmotic adjustment under osmotic stress. Jiang et al. [59] found that higher concentrations of metabolites related to amino acids were observed in melatonin-treated plants than in control, similar to the present study. Similarly, the metabolites involved in carbon metabolism were increased by melatonin treatment [60,61,62].

Transcriptomic analysis revealed that genes engaged in different metabolite pathways, signal transduction, transcription factors, and kinase activity were upregulated in response to stress. Genes involved in energy metabolism encoding mitochondrial ATPase, cytochrome c, ferredoxin, sucrose, and starch metabolism were identified in DEGs between melatonin-treated and untreated plants [63,64]. Earlier findings of Zhao et al. [65] confirmed that applying melatonin increases the expression level of genes involved in signal transduction and carbon metabolism of plants under stress, thereby improving the photosynthetic process. The MAP kinase pathway controls the melatonin-mediated regulation of plant response to stress by activating either H_2_O_2_ or Ca^2+^-dependent pathways [66,67,68].

Yield traits like the number of pods per plant, number of seeds per pod, pod length, the total number of seeds per plant, total pod weight, seed yield per plant, 100 seed weight, and harvest index were found to be higher in melatonin-treated plants in comparison with control plants. Overall application of melatonin increased the yield under stress compared to that of unsprayed plants [69,70]. The increase in yield could be associated with increased sink strength [71], hormonal balance [72], and less oxidative damage [73]. An increase in seed yield due to foliar application of melatonin was proved in soybean and maize [74,75].

## 4. Materials and Methods

### 4.1. Plant Material and Growth Conditions

An experiment was conducted during 2021–2022 at the glasshouse and open-top chamber at the Department of Crop Physiology, Tamil Nadu Agricultural University, Coimbatore. Seeds of mung bean var. CO 8 were used in this study. The pot culture experiment was conducted with five treatments in a completely randomized block design (CRD). The soil mixture was red soil, sand, and vermicompost in a ratio of 3:1:1, and 20 kg of the soil mixture was filled in each pot. The seeds were surface sterilized with 3% sodium hypochlorite for two minutes and washed thrice with distilled water; then, the seeds were treated with melatonin at 100 µM for 6 h and air dried. A separate set of melatonin-treated and untreated seeds for stress and control treatments were sown directly in pots. The recommended dose of fertilizer was applied to the crop. The plants were allowed to grow under normal conditions until the flowering stage for stress imposition.

### 4.2. Treatment Details and Stress Imposition

Treatments included the exogenous application of 100 µM melatonin as a seed treatment, foliar spray, and a combination of both seed treatment and foliar spray; absolute control (no spray and ambient condition) and control (stress without melatonin treatment) were also included in the treatments to evaluate the efficiency of melatonin under stress conditions. At the flowering stage, stress was imposed for ten days (31st to 40th day), and a foliar melatonin spray was carried out on the third day (33rd day). Drought stress was induced by withholding water, and soil moisture content was measured daily using an ML2 Theta Probe moisture meter (Delta-T Soil moisture kit, Model: SM150, Delta-T Devices, Cambridge). The high-temperature stress experiment was conducted in an open-top chamber (OTC). Using an infrared heater, the chambers’ temperature was gradually increased from 9.00 am to 5.00 pm to the desired level above the ambient temperature [76]. The average ambient temperature that prevailed during high-temperature stress was 32 °C, and the temperature was elevated to 34 °C (32 °C + 2 °C) and 36 °C (32 °C + 4 °C) inside the OTC. Likewise, in combined drought and high-temperature stress, the average ambient temperature was 34 °C, and stressed and melatonin-treated plants were exposed to elevated temperatures (ambient + 2 °C and ambient + 4 °C), resulting in 36 °C and 38 °C. The temperature and relative humidity of the OTC were continuously monitored using wireless sensors.

### 4.3. Chlorophyll Index (SPAD Value)

The chlorophyll index was measured using a SPAD meter designed by the Soil Plant Analytical Development (SPAD) section, Minolta, Japan. The Minolta SPAD-502 measures chlorophyll content as the ratio of transmittance of light at a wavelength of 650 nm and 940 nm. In every treatment, three readings were taken from each replication, and in each leaf, readings were taken from the top, middle, and bottom of the leaf. Finally, the average value was computed using the method described by Minolta [77] and Monje and Bugbee [78].

### 4.4. Chlorophyll Fluorescence

The leaf samples were dark adapted for 20 min, and the chlorophyll fluorescence was computed using the portable chlorophyll fluorometer (Model-OS1p040111 Advanced, Opti-Sciences, Hudson, NH, USA). The key fluorescence parameters, viz. Fo (initial fluorescence) and Fm (maximal fluorescence), were measured, and the ratio of Fv/Fm was calculated [79].

### 4.5. Leaf Gas Exchange Parameters

Gas exchange parameters, viz. photosynthetic rate, transpiration rate, and stomatal conductance, were recorded using a portable photosynthesis system (LI-6400 XT, LiCORInc., Lincoln, NE, USA). The readings were taken from 10.00 am to 12.00 noon on a clear sunny day. The photosynthetically active radiation was set at 1500 µmol photons m^−2^ s^−1^, and the CO_2_ level was set at 410 ppm. A fully expanded third leaf from the top was used for measuring the gas exchange traits. The photosynthetic rate was expressed as µmol CO_2_ m^−2^ s^−1^, stomatal conductance was expressed as mol H_2_O m^−2^ s^−1^, and transpiration rate was expressed as mmol H_2_O m^−2^ s^−1^.

### 4.6. Hydrogen Peroxide (H_2_O_2_)

H_2_O_2_ present in the leaves was visually identified with the 3,3-diamino-benzidine (DAB) staining technique [80]. The leaves were incubated overnight in DAB solution (1 mg mL^−1^ DAB, 5 mM Na_2_HPO_4,_ and 0.05% Tween 20 at pH 3.8). After incubation, the leaves were destained with a destaining solution (ethanol:acetic acid in the ratio of 3:1). Development of brown color representing H_2_O_2_ accumulation in the leaves was examined under a Leica microscope, and an image was captured.

### 4.7. Superoxide Radical (O_2_^−^)

The nitro blue tetrazolium chloride (NBT) staining method was followed to detect superoxide radicals [80]. The leaves were immersed in the staining solution (0.5 mg mL^−1^ NBT, 50 mM sodium phosphate buffer at pH 7.5) overnight. Later, the leaves were decolorized using a destaining solution (ethanol: acetic acid in a ratio of 3:1). Superoxide radicals were identified by the development of blue color, and an image was captured.

### 4.8. Malondialdehyde

The malondialdehyde (MDA) content was assessed using the thiobarbituric acid (TBA) reaction according to the method illustrated by Karabal et al. [81]. First, 0.5 g of leaf tissue was homogenized with 5 mL of 0.1% trichloroacetic acid (TCA) and centrifuged at 10,000 rpm for 5 min. About 4 mL of 20% trichloroacetic acid (TCA) containing 0.5% thiobarbituric acid (TBA) was added to 1 mL of aliquot and heated at 95 °C for 30 min. Immediately, the tubes were cooled and centrifuged at 10,000 rpm for 10 min. The supernatant was collected, and absorbance was measured at 532 and 600 nm and expressed as nmol g^−1^ of fresh weight.

### 4.9. Electrolyte Leakage

The electrolyte leakage was determined by the method mentioned by Zhang et al. [82]. Twenty-five leaf bits 2 cm^2^ in size were transferred into 10 mL of deionized water, and the initial electrical conductivity was recorded as EC_0_. Then, the samples were subjected to 25 °C for one hour, and electrical conductivity was noted as EC_1_. Finally, the samples were autoclaved at 100 °C for 10 min, and the final electrical conductivity was measured as EC_2_. The electrolyte leakage was computed using the following formula and expressed as a percentage:

Electrolyte leakage = [(EC_1_ − EC_0_)/(EC_2_ − EC_0_)] × 100

### 4.10. Antioxidant Enzyme Activity

To estimate the activity of antioxidant enzymes like superoxide dismutase, catalase, and ascorbate peroxidase, an enzyme extract was prepared by weighing 0.5 g of leaf sample and grinding it into powder using liquid nitrogen. The enzyme extract was prepared by homogenizing the leaf powder with 0.1 M phosphate buffer (pH 6.8) containing 0.1 mM EDTA and 1% Polyvinylpyrrolidone (PVP) in a pre-chilled pestle and mortar. The collected supernatant was used to estimate the activity of antioxidant enzymes, viz. SOD and CAT. To estimate ascorbate peroxidase, 1 mM ascorbate was added to extract the enzyme, and the homogenate was centrifuged at 10,000 rpm for 30 min at 4 °C.

#### 4.10.1. Superoxide Dismutase

The SOD activity was examined according to the method of Dhindsa et al. [83]. The reaction mixture consisted of 1.3 µM riboflavin,13 mM methionine, 63 µM nitro blue tetrazolium chloride (NBT), 0.05 M sodium carbonate, 1% Triton X-100, 50 mM sodium phosphate buffer (pH 7.8), and enzyme extract, and the final volume was made up to 3 mL by using distilled water. Test tubes were kept under illumination for color development, whereas the non-illuminated reaction mixture without enzyme extract served as a blank. The SOD activity was determined as the amount of enzyme required to cause 50% inhibition of the reduction of NBT and expressed as enzyme units mg protein^−1^ min^−1^.

#### 4.10.2. Catalase

Catalase activity was analyzed using a method described by Hugo and Lester [84]. About 0.5 mL of 75 mM H_2_O_2_, 1.5 mL of 0.1 M phosphate buffer (pH 7), and 50 µL of enzyme extract were added; finally, the total reaction mixture volume was made up to 3 mL by adding distilled water. The addition of H_2_O_2_ started the reaction. The decrease in absorbance at 240 nm was recorded for 1 min at 15 intervals, and enzyme activity was computed by calculating the amount of H_2_O_2_ decomposed. For catalase activity, the extinction coefficient of 39.4 mM^−1^ cm^−1^ was used, and activity was expressed as µg of H_2_O_2_ reduced mg protein^−1^ min^−1^.

#### 4.10.3. Ascorbate Peroxidase

Ascorbate peroxidase activity was determined according to the method of Nakano and Asada [85]. The reaction mixture for ascorbate peroxidase contained 50 mM potassium phosphate (pH 7.0), 0.5 mM ascorbate, 0.1 mM EDTA, enzyme extract, and 0.1 mM H_2_O_2_. The final total volume was made up to 3 mL. The reaction was started by the addition of H_2_O_2_. The absorbance decrease at 290 nm was recorded for 3 min at 30 s intervals. For APX activity, the extinction coefficient was 2.8 mM^−1^ cm^−1^, and activity was expressed in terms of change in OD at 430 nm g^−1^ min^−1^.

### 4.11. Proline

Proline content was determined by macerating the leaf sample with 10 mL of 3% sulfosalicylic acid and centrifuging it at 3000 rpm for 10 min. In test tubes, 2 mL of each supernatant, acid ninhydrin, glacial acetic acid, and orthophosphoric acid were added and kept in a water bath for 1 h. After cooling, the contents were transferred to a separating funnel, and 4 mL of toluene was added. Subsequently, it was shaken for 30 s, and the colored solution was measured at 520 nm in a UV spectrophotometer [86] and expressed as mg g^−1^.

### 4.12. Metabolomics Profiling

The leaf samples of different treatments exposed to drought and high-temperature stress were collected for metabolite profiling, and the extractant was analyzed by GC-MS technique [87]. Leaf samples of approximately 0.3 g were collected and homogenized with liquid nitrogen. Then, 1 mL of methanol was added and ground. Then, it was incubated in a thermomixer incubator at 850 rpm at 70 °C for 30 min. The contents were centrifuged at 14,000 rpm for 10 min, and the supernatant was collected and filtered through a 0.45 μm membrane syringe filter. Derivatization was performed by adding 50 µL of methoxyamine hydrochloride (20 mg mL^−1^ in pyridine) and vortexing for 30 s. After, it was incubated at 37 °C in a thermomixer incubator at 500 rpm for 2 h. After that, 80 µL of MSTFA was added and incubated for 30 min at 37 °C. The supernatant was centrifuged at 12,000 rpm at 4 °C for 10 min. Finally, the supernatant was subjected to GC-MS (Shimadzu, Canby, OR, USA) analysis. 

For GC-MS, 1 µL of the derivatized extract was introduced into a DB-5MS capillary (30 × 0.25 × 0.25 µm) column. The temperature of the inlet was set at 260 °C. After a six min solvent delay, the initial GC oven temperature was set at 70 °C. After 1 min of injection, the GC oven temperature was raised to 280 °C at 15 °C per min and held at 280 °C for 15 min. The injection temperature was set to 240 °C, and the ion source temperature was matched. Helium was the carrier gas with a constant flow rate of 1mL per min. The measurement was performed with electron impact ionization (70 eV) in the full scan mode (*m*/*z* from 30 to 550). The metabolites were identified based on retention time index specific masses via comparing with reference spectra in mass spectral libraries (NIST 2005, Wiley 7.0).

### 4.13. Transcriptomic Analysis

Mung bean leaf samples exposed to drought and high-temperature stress during the reproductive stage were collected. Later, the leaf samples were immersed in RNA solution to maintain RNA stability and integrity. The study of the differential expression pattern of genes included total RNA extraction, library preparation, sequencing, pre-processing of RNA-Seq reads, reference mapping of RNA-Seq reads with *Vigna radiata* (L.) HISAT genome, and analysis of differentially expressed genes. Two replications for each treatment were maintained. Total RNA was extracted from all six samples using the RNAeasy Plant mini kit (Qiagen, Redwood City, CA, USA) according to the manufacturer’s instructions after treatment with RNase-free DNase I to eliminate genomic DNA. The concentration and integrity of the extracted RNA were assessed with a Thermo Scientific NanoDrop 8000 Spectrophotometer and Agilent 2100 Bioanalyzer, respectively (Agilent Technologies, Santa Clara, CA, USA). Raw data (raw reads) of the FASTQ format were processed by fastqc. The cDNA library was prepared with ~20 ng of total RNA according to the Illumina TrueSeq RNA Sample Preparation Kit (Illumina) protocol. The library was then amplified, the final library yield was recorded, and the resulting library was subjected to the paired-end sequence. High-quality reads were quantified for transcript abundance by defining the “transcripts per million” (TPM) value for each transcript using Salmon v1.14.0 (https://combine-lab.github.io/salmon (accessed on 12 March 2023)), using genome and transcriptome sequences downloaded from “Ensembl” database (https://plants.ensembl.org/index.html, accessed on 12 March 2023)) for reference and indexing.

### 4.14. Yield Parameters

Yield components like the number of pods per plant, number of seeds per pod, pod length, total number of seeds per plant, total pod weight, seed yield per plant, 100 seed weight, and harvest index were recorded.

### 4.15. Statistical Analysis

The design of the experiment was a completely randomized design (CRD) with four replications, and the data collected for various traits were statistically analyzed by using R software (version 4.1.2) with analysis of variance (ANOVA). Critical difference (CD) was computed at a five percent probability (*p* ≤ 0.05).

## 5. Conclusions

**Figure 18:**
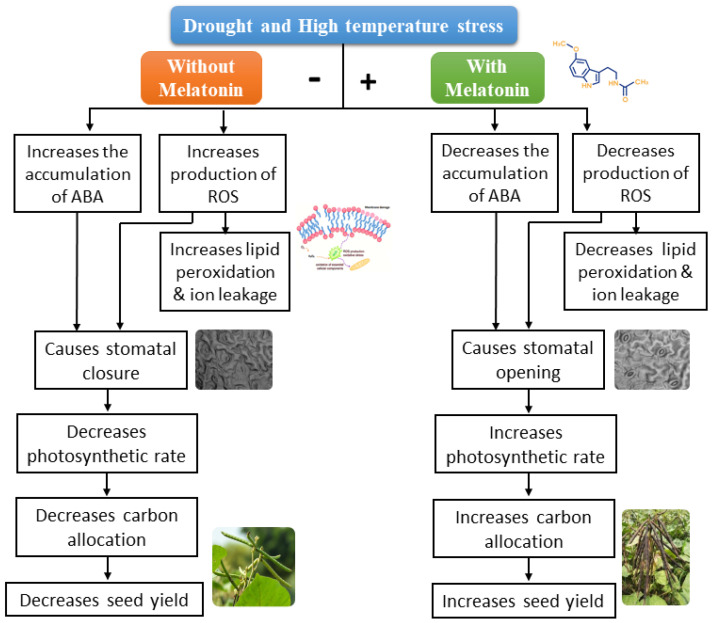
Schematic representation of melatonin-mediated drought and high−temperature stress tolerance in plants.

In summary, drought, high temperature, or combined drought and high-temperature stress during the reproductive stage showed a pronounced negative effect on the physiological and biochemical processes of the plant, which in turn was reflected in the yield of mung bean. Drought and high-temperature stress affect the membrane integrity through increased ROS generation. The seed treatment plus foliar application of 100 µM melatonin alleviates the ill effects of drought and high-temperature stress. It enhanced physiological processes such as chlorophyll index, PSII efficiency, and gas exchange parameters. In addition, the application of melatonin significantly reduced lipid peroxidation and ROS production through enhanced antioxidant enzyme activity (SOD, CAT, and APX) under individual and combined stress due to drought and high temperature (Figure 18). Hence, melatonin is recommended as an appropriate management strategy to sustain the potential yield of mung bean under stress conditions.

## Data Availability

Not applicable.
